# Availability and use of cancer decision-support tools: a cross-sectional survey of UK primary care

**DOI:** 10.3399/bjgp19X703745

**Published:** 2019-05-08

**Authors:** Sarah Price, Anne Spencer, Antonieta Medina-Lara, Willie Hamilton

**Affiliations:** Cancer Diagnosis (DISCO) Group, Institute of Health Research, College of Medicine and Health, University of Exeter, Exeter.; Institute of Health Research, College of Medicine and Health, University of Exeter, Exeter.; Institute of Health Research, College of Medicine and Health, University of Exeter, Exeter.; Cancer Diagnosis (DISCO) Group, Institute of Health Research, College of Medicine and Health, University of Exeter, Exeter.

**Keywords:** computer-assisted decision making, cross-sectional survey, decision support systems, early detection of cancer, primary health care

## Abstract

**Background:**

Decision-support tools quantify the risk of undiagnosed cancer in symptomatic patients, and may help GPs when making referrals.

**Aim:**

To quantify the availability and use of cancer decision-support tools (QCancer^®^ and risk assessment tools) and to explore the association between tool availability and 2-week-wait (2WW) referrals for suspected cancer.

**Design and setting:**

A cross-sectional postal survey in UK primary care.

**Methods:**

Out of 975 UK randomly selected general practices, 4600 GPs and registrars were invited to participate. Outcome measures included the proportions of UK general practices where cancer decision-support tools are available and at least one GP uses the tool. Weighted least-squares linear regression with robust errors tested the association between tool availability and number of 2WW referrals, adjusting for practice size, sex, age, and Index of Multiple Deprivation.

**Results:**

In total, 476 GPs in 227 practices responded (response rates: practitioner, 10.3%; practice, 23.3%). At the practice level, 83/227 (36.6%, 95% confidence interval [CI] = 30.3 to 43.1) practices had at least one GP or registrar with access to cancer decision-support tools. Tools were available and likely to be used in 38/227 (16.7%, 95% CI = 12.1 to 22.2) practices. In subgroup analyses of 172 English practices, there was no difference in mean 2WW referral rate between practices with tools and those without (mean adjusted difference in referrals per 100 000: 3.1, 95% CI = −5.5 to 11.7).

**Conclusion:**

This is the first survey of cancer decision-support tool availability and use. It suggests that the tools are an underused resource in the UK. Given the cost of cancer investigation, a randomised controlled trial of such clinical decision-support aids would be appropriate.

## INTRODUCTION

Diagnosing cancer quickly after patients develop symptoms is a UK priority.^1^ National guidelines help UK GPs select which patients warrant referral and investigation for suspected cancer.^2^^–^4 This ‘gatekeeper’ system may cause diagnostic delay;^5^ for example, 2-week-wait (2WW) referrals for suspected cancer are less likely when patients present with ‘low-risk but not no-risk’ than when they have ‘alarm’ symptoms.^6^ Clinical decision-support tools for cancer quantify the risk of an undiagnosed cancer in symptomatic patients.^7^ Two main types are available: risk assessment tools (RATs) and QCancer^®^.^8^^,^^9^ RATs are available for 18 specific cancer sites, and use symptoms and test results to estimate the risk of cancer.^8^^,^^10^^–^17 QCancer uses symptoms, test results, and patient risk factors for six specific cancer sites,^9^^,^^18^^–^22 plus one for each sex, estimating the overall risk of cancer.^23^^,^^24^ RATs were distributed to all 10 000 general practices in England in 2012 as mouse mats and flipcharts and QCancer is freely accessible on the internet (https://qcancer.org).

In 2013, both RATs and QCancer were incorporated into GP software systems and renamed collectively as ‘electronic clinical decision-support tools for cancer’. For simplicity, all tools are hereafter called paper or electronic ‘cancer tools’. RATs were integrated into the GP software system Vision (INPS) and QCancer into EMIS Web (Egton Medical Information Systems). Together, EMIS Web and Vision had 62% of the market share of GP IT systems in 2015.^25^

There is little research on the clinical utility of cancer tools, or on their availability and uptake in UK primary care.^7^ A recent qualitative study of a convenience sample of 126 GPs aimed to improve the understanding of how GPs use cancer tools. The study reported that 18.3% of GPs used either RAT or QCancer, but that overall awareness of these tools was low (Chisnell *et al* unpublished data, 2017). A cohort study compared the numbers of cancer investigations and diagnoses before and after the introduction of colorectal and lung RATs to 165 general practices in England.^26^ The introduction of RATs was associated with increased diagnostic activity and additional diagnoses of lung and colorectal cancer.^26^ A 2 × 2 design trial of a GP intervention, which included the colorectal and lung RATs, found no evidence that it was associated with faster time to diagnosis of cancer in rural Australia.^27^ No studies have investigated the association between use of cancer tools and use of the UK’s urgent referral pathway for suspected cancer. Understanding this association is important for two reasons: the impact of increased referrals on resources; and use of the 2WW referral pathway is associated with improved cancer outcomes.^28^

**How this fits in**

Clinical decision-support tools quantify the risk of an undiagnosed cancer in symptomatic patients and may help GPs to improve their selection of patients for investigation of suspected cancer. The tools are an integral part of the National Cancer Strategy, yet their uptake in general practice is unknown. The survey studied here — the first of the availability and use of cancer decision-support tools in the UK — reports that the paper-based and electronic tools are available to GPs in approximately one-third (36.6%, 95% CI = 30.3 to 43.1) of UK practices and likely to be used in 16.7% (95% CI = 12.1 to 22.2).

Therefore, the primary aims of this study were to identify the proportions of general practices and of GPs with access to cancer tools, and, where there is access to tools, the proportion of practices that actually use them. The secondary aim was to investigate any association between a practice’s access to cancer tools and referral activity for suspected cancer.

Two main measures of cancer referral activity are available in the Public Health England dataset. The first is a diagnostic process indicator — the age- and sex-standardised number of referrals adjusted for practice size — and is reliable at the practice level.^29^ This indicator is suitable for assessing whether use of cancer tools is associated with increased numbers of referrals and the potential impact on resources. The second is a diagnostic outcome indicator: the proportion of patients undergoing a 2WW referral who are subsequently diagnosed with cancer (conversion rate). However, the small numbers of cancers diagnosed per practice make this measure unreliable,^29^ so the authors decided against using it as an outcome measure for investigating the association between use of tools and cancer outcomes.

## METHOD

The present study was a cross-sectional postal survey in UK primary care. The questionnaire was planned using the best-practice guidelines for survey design,^30^ and was further reviewed and edited by the originators of RATs (Willie Hamilton) and QCancer (Julia Hippisley-Cox and Carol Coupland). Images of the paper-based tools and screenshots of the electronic cancer tools were included to ease their identification. Questionnaires included a general practice identifier, but not the name of the responding GP. The questionnaire was piloted with five GPs for its clarity and design. To measure tool usage, GPs were asked how likely they would be to consult desktop or electronic tools in a patient with symptoms of possible cancer, using a 4-point Likert scale: very likely, likely, unlikely, and very unlikely.^31^ Participants were asked to select any aspects of the tools they found helpful, from a list of positive aspects of lung and colorectal cancer RATs reported previously.^26^^,^^32^^,^^33^ Participants were also asked to rank in order of usefulness the three main interactive functions of the electronic cancer tools: ‘alert/prompt’ where cancer risk scores appear automatically once a patient’s electronic notes are opened if there is a risk of any individual cancer ≥2%; ‘symptom checker’ where GPs can request a patient’s cancer risk; and ‘searches/report’ where GPs can search records and produce summaries of patients ranked by cancer risk. The questionnaire, covering letter, and information sheet are available from the authors on request. The questionnaire had no free-text comments section; however, any written comments, or comments sent by email or phone, were recorded (a list of comments received is available from the authors on request).

The survey was administered by a commercial firm, Binley’s. It was conducted at the practice level, reflecting how practice software decisions are generally made. The invited population was general practices in the UK and clinically active GP partners or principals, sessional GPs (including salaried and locum GPs), and GP registrars. Assuming a population proportion of 50% of practices with access to a tool and adjusting for the clustered design, the authors estimated that a sample size of 392 general practices was required at the 95% confidence level for 5% precision. The authors estimated a 40% response rate, so obtained a random probability sample of 975 general practices from Binley’s.^34^ Questionnaires were sent to all GPs (*n* = 4350) and registrars (*n* = 250) in these practices in July 2017, with a follow-up questionnaire 1 month later for practices that did not respond. Data collection was stopped 14 weeks after reminders were issued. To incentivise participation, a charitable donation of £7.50 (to Cancer Research UK and Macmillan Cancer Support equally) was made for the first 400 replies.

### Analyses

If any single GP reported that they had access to cancer tools, it was assumed that this was possible for all other GPs at that practice. The authors used simple descriptive statistics for access to and use of cancer tools. For completeness, GP and practice-level responses are reported. Survey responses from English practices were linked to the Public Health England dataset. Referral activity was measured using the practices’ age- and sex-adjusted numbers of 2WW referrals for suspected cancer per 100 000 of the population.^29^ The association between practice tool availability and 2WW referral rate was estimated using weighted least-squares regression with robust errors, adjusted for the practice’s index of multiple deprivation.^35^^–^38

## RESULTS

### Participant characteristics

Responses were received from 473 GPs and three GP registrars in 227 practices. The response rate at the practitioner level was 10.3% and 23.3% at the practice level. Responding practices had a median of 6 GPs (interquartile range [IQR] 4–8), of whom a median of 2 (IQR 1–3) responded. The mean within-practice response rate was 43.7%, 95% confidence interval [CI] = 39.3 to 48.1). Unprompted comments indicated that lack of time (*n* = 12) and lack of awareness of the tools (*n* = 6) were the most common reasons for non-response (a list of comments received is available from the authors on request). Of the responders, 294 (61.8%) had been practising for ≥11 years; 299 (62.8%) were working between five and eight sessions per week (Appendix 1). EMIS Web was the most frequently used IT software (96/227, 42.3%), followed by TPP SystmOne (74/227, 32.6%) and INPS Vision (32/227, 14.1%), largely matching the national market share of these software packages (Appendix 2). The distribution of practices by Index of Multiple Deprivation was broadly representative of practices in the UK (data are available from the authors on request).

Access to a paper-based cancer tool in mouse mat or flip chart form was reported by 63 of the 476 (13.2%) GPs and registrars (Table 1). At the practice level, tools were available in 51 of the 227 (22.5%, 95% CI = 17.2 to 28.5) practices. A list of ‘other’ tools used is available from the authors on request, and consists of national guidelines or summaries thereof, which do not quantify the risk of undiagnosed cancer. Of the 63 GPs with access to a mouse mat or flip chart, *n* = 39 (61.9%) reported that they were unlikely or very unlikely to use it during a consultation with a patient with possible symptoms of cancer (Table 1). The participants’ choices from a selected list of helpful aspects of the paper-based cancer tools are reported in Table 2.

**Table 1.** Responses to questions asking about availability and likely use of paper-based risk assessment tools (*N* = 476)

**Risk assessment tool format**	**GPs and registrars, *n* (%)**
**Mouse mat or flip chart**	63 (13.2)
**Likelihood of using mouse mat or flip chart**	
Very likely	5 (7.9)
Likely	14 (22.2)
Unlikely	29 (46.0)
Very unlikely	10 (15.9)
Missing/not answered	5 (7.9)

**Other**	30 (6.3)

**None of these**	326 (68.5)

**Missing/not answered**	57 (12.0)

**Table 2.** Functions of the paper and electronic clinical decision-support tools considered useful

**Function considered useful**	**GPs and registrars reporting function is useful in tools**	

	**Paper-based, *n* (%) (*N*= 63)**	**Electronic, *n* (%) (*N*= 58)**
Assessing cancer risk in patients with:		
Non-specific symptoms	28 (44)	20 (35)
Multiple symptoms	25 (40)	21 (36)

Discussing cancer risk with a patient	19 (30)	17 (29)

Increasing the awareness of cancer as a possible diagnosis	17 (27)	13 (22)

Reassuring anxious patients	16 (25)	10 (17)

Prompting referrals that would otherwise not have been made	13 (21)	8 (14)

Increasing the certainty of clinical decision making	25 (40)	7 (12)

Increasing the awareness of cancer symptoms	10 (16)	7 (12)

Discussing investigation with symptomatic patients	9 (14)	3 (5)

None of these	8 (13)	17 (29)

The electronic cancer tool was downloaded or activated on the IT system of 58 of 476 GPs and registrars (12.2%) (Table 3), equating to a practice level of 42/227 (18.5%, 95% CI = 13.6 to 24.2) (Table 4). Of the 476 GPs and registrars, 174 (36.6%) were unaware of electronic tools, and 39 (8.2%) reported that they would like to have them but that they are not available for their system (Table 3).

**Table 3.** Responses to question asking about electronic clinical decision support tool availability (*N* = 476)

**Option**	**GPs and registrars, *n* (%)**
Unaware of eCDS	174 (36.6)
eCDS is downloaded/activated for my IT system	58 (12.2)
eCDS is available for my IT system, but my practice has not downloaded/activated it	23 (4.8)
eCDS is available for my IT system, and my practice has plans to download/activate it in future	6 (1.3)
To my knowledge, eCDS is not available for my IT system	108 (22.7)
eCDS is not available for my IT system but I would like to have it	39 (8.2)
Missing/not answered	68 (14.3)

*eCDS* = *electronic clinical decision support.*

**Table 4.** General practices: IT software and cancer tools

	**Cancer tools are downloaded/activated, *n* (%)**		
**General practice IT software**	***N***	**No**	**Yes**
EMIS Web (QCancer^®^)	96	64 (66.7)	32 (33.3)
EMIS LV^a^	5	5 (100.0)	0 (0.0)
EMIS PCS^a^	11	11 (100.0)	0 (0.0)
INPS Vision (RAT)	32	22 (68.8)	10 (31.2)
TPP SystmOne ^a^	74	74 (100.0)	0 (0.0)
Microtest^a^	3	3 (100.0)	0 (0.0)
Other^a^	6	6 (100.0)	0 (0.0)
Total	227	185 (81.5)	42 (18.5)

a *Practices that did not use either EMIS Web or INPS Vision could not have had access to electronic clinical decision-support tools. RAT* = *risk assessment tool.*

Of the 58 GPs with access to the electronic cancer tools, 17 (29.3%) reported having integrated it into their practice, and nine (15.5%) having received training. Only five GPs had both received training and had integrated the tool into their practice. At the practice level, training had been received by at least one GP in six (14.3%) practices with access to the tool. The tool was integrated into the practice of at least one GP in 15 (35.7%) practices.

The ‘alert prompt’ and ‘symptom checker’ functions were deemed the most useful by 16 (27.6%) and 14 (24.1%) of the 58 GPs with access to the tool, respectively. Two-thirds (39/58, 67.2%) reported that they would be unlikely or very unlikely to use an electronic cancer tool to assess a patient whose symptoms may represent cancer. The participants’ choices from a selected list of helpful aspects of the electronic cancer tools are reported in Table 2.

Overall, of the 476 GPs, 112 (23.5%, 95% CI = 19.7 to 27.6) had access to a cancer tool in either paper (*n* = 54) or electronic (*n* = 49) format, or both (*n* = 9).

At the practice level, this equates to at least one GP with access in 83 practices (36.6%, 95% CI = 30.3 to 43.1). Of the 227 general practices, 38 (16.7%, 95% CI = 12.1 to 22.2) contained at least one GP who had access to the tools and was likely or very likely to use them. Practices using EMIS Web and INPS Vision were equally likely to have downloaded/activated the software (32/96, 33.3% EMIS Web, 10/32, 31.3% INPS Vision) (Table 4).

### Association between use of tools and 2WW referral activity

Of the 172 practices in England with published 2WW referral and conversion rates, 68 had access to either a paper or electronic cancer tool. There was no difference in mean 2WW referral rate between practices with or without access to either type of tool, after adjusting for Index of Multiple Deprivation (mean difference 3.1 referrals per 100 000 [95% CI = −5.5 to 11.7] per 100 000, *P* = 0.478) (Table 5).

**Table 5.** Regression analysis output. Dependent variable sex- and age-adjusted urgent 2-week wait referrals per 100 000 population (*N* = 172; *R*^2^ = 0.0635)

	**Referral rate per 100 000 population**		
**Variable**	**Mean difference**	**95% CI**	***P-*value**
Availability of tool (yes/no)	3.1	−5.5 to 11.7	0.478
Index of Multiple Deprivation	0.6	0.1 to 1.0	0.010

a CI = confidence interval.

## DISCUSSION

### Summary

This is the first UK-wide survey of the availability of cancer tools. These tools, in paper or electronic format, were available to GPs in approximately one-third of UK practices. The proportion of general practices where at least one GP had access to the tools and was likely or very likely to use them was 16.7% (95% CI = 12.1% to 22.2%). There are no current plans to re-release paper-based tools, with the expectation that the electronic version will become the norm. Therefore, 18.5% of general practices with access to the electronic version may be the more important measure. Currently, the tools are only available via EMIS Web and INPS Vision, and approximately one-third of the practices using these software systems had opted to download or activate them. The software will shortly be integrated into TPP SystmOne, with approximately 33% of the UK market share. Between them, EMIS Web, TPP SystmOne, and INPS Vision represent over 95% of the GP software systems available;^25^ therefore, in the near future it is reasonable to assume that nearly all GPs could access tools, should they choose to download or activate them.

It could be argued that use of the tools risks overwhelming secondary care resources; however, the authors found no evidence of an association between tool availability and an increase in the number of 2WW referrals at the practice level. The inability to find differences may be because the tools have only been available for a short while and are not yet embedded in clinical practice. To assess the effectiveness of the tools, future studies will need to consider the 2WW referrals and the impact these have on stage at diagnosis and survival. The present study’s finding that the tools are an underused resource in the UK suggests that there is potential to explore the effectiveness of these tools on appropriate referrals to improve cancer outcomes within a randomised controlled trial.

### Strengths and limitations

The selection of a 40% response rate had seemed reasonable in the present study, based on a reported value of 61% (95% CI = 59 to 63) in 2011, and adjusted downward to reflect the current workload crisis in general practice.^34^^,^^39^ However, the authors’ achieved sample was smaller than planned, resulting in wide CIs. The low response rate probably reflects high GP workload, as volunteered by practice managers and reported elsewhere^39^ (Chisnell *et al* unpublished data, 2017). Responder bias is important to consider, given the present study’s low response rate. The study presented here would overestimate tool availability if responders were more likely than non-responders to have access to the tools. However, the proportion of practices with computer systems supporting electronic tools was not overrepresented in the authors’ sample: 57% of responding practices had INPS Vision and EMIS Web systems, which was very similar to the national picture of 62%. This suggests that the response rate is unrelated to access to tools via the software used at the practice, and that the effect of responder bias on the estimates of tool availability and use is likely to be small. The possibility remains that responses to questions about use of the tools may have been influenced by GPs’ cognitive biases.

Furthermore, it could be argued that practices that have chosen to access the cancer tools are more engaged in the early cancer diagnosis framework than practices who have not. This might be expected to lead to overestimates of the association between use of the tools and the number of 2WW referrals.

### Comparison with existing literature

There is no comparable literature on practice-level availability and use of cancer tools for cancer in the UK or elsewhere. Chisnell *et al* (unpublished data, 2017) reported that use of cancer tools was low, but this estimate is at the GP level.

The authors’ finding of low-level use of cancer tools is supported by qualitative studies reporting that the cancer tool’s screen alerts increase the risk of disuse through ‘prompt fatigue’,^32^^,^^33^ and generally low levels of awareness (Chisnell *et al* unpublished data, 2017).

### Implications for research and practice

This study and previous qualitative work suggest that improvements in design and training of tools may increase uptake.^26^^,^^33^^,^^40^ Any training should encourage GPs to maximise symptom recording in a patient’s medical record, using a code rather than text fields. This is because the algorithms rely on coded data, and omission of data recorded in text fields is associated with bias.^41^

As the levels of tool uptake are relatively low, it remains possible to carry out a randomised controlled trial to assess whether these tools are genuinely helpful in improving the selection of patients for investigation and to assess the impact on resource use in a cost-effectiveness framework. The potential benefits of improved patient selection include better targeting of investigation resources, earlier diagnosis, and reduced treatment costs.^26^^,^^40^^,^^42^^–^47 Such a trial should include a study of barriers to use, and ways to overcome them.

## Appendix 1. Responding GP and registrar demographics: numbers of years in practice and number of consultation sessions worked per week (*N* = 476)

**Table:**

**Demographic information**	**GPs and registrars, *n***	**%**
**Time in practice, years**		
<1	20	4.2
1–5	57	12.0
6–10	57	12.0
11–20	145	30.5
21–30	111	23.3
>30	38	8.0
Missing	48	10.1

**Consultation sessions per week, *n***		
1–2	10	2.1
3–4	70	14.7
5–6	140	29.4
7–8	159	33.4
9–10	40	8.4
>10	5	1.0
Missing	52	10.9

## Appendix 2. General practice software (*N* = 227)

**Table:**

**GP and registrar IT software (eCDS tool)**	**GPs and registrars, *n* (%)**	**National market share, %**
EMIS Web (QCancer^®^)	96 (42.3)	52.4
TPP SystmOne^a^	74 (32.6)	33.0
INPS Vision (RAT)	32 (14.1)	9.9
EMIS PCS^a^	11 (4.9)	0.01
EMIS LV ^a^	5 (2.2)	0.02
Other^a^	6 (2.6)	3.3
Microtest^a^	3 (1.3)	1.4

a *Practices that did not use either EMIS Web or INPS Vision could not have had access to electronic clinical decision-support tools. eCDS* = *electronic clinical decision support.*
